# Alkaliphilic Bacteria with Impact on Industrial Applications, Concepts of Early Life Forms, and Bioenergetics of ATP Synthesis

**DOI:** 10.3389/fbioe.2015.00075

**Published:** 2015-06-03

**Authors:** Laura Preiss, David B. Hicks, Shino Suzuki, Thomas Meier, Terry Ann Krulwich

**Affiliations:** ^1^Department of Structural Biology, Max Planck Institute of Biophysics, Frankfurt, Germany; ^2^Department of Pharmacology and Systems Therapeutics, Icahn School of Medicine at Mount Sinai, New York, NY, USA; ^3^Geomicrobiology Group, Kochi Institute for Core Sample Research, Japan Agency for Marine-Earth Science and Technology, Nankoku, Japan; ^4^Microbial and Environmental Genomics, J. Craig Venter Institutes, La Jolla, CA, USA

**Keywords:** alkaliphiles, biotechnology, serpentinization, ATP synthase, *Bacillus pseudofirmus* OF4, bioenergetics, proton-motive force

## Abstract

Alkaliphilic bacteria typically grow well at pH 9, with the most extremophilic strains growing up to pH values as high as pH 12–13. Interest in extreme alkaliphiles arises because they are sources of useful, stable enzymes, and the cells themselves can be used for biotechnological and other applications at high pH. In addition, alkaline hydrothermal vents represent an early evolutionary niche for alkaliphiles and novel extreme alkaliphiles have also recently been found in alkaline serpentinizing sites. A third focus of interest in alkaliphiles is the challenge raised by the use of proton-coupled ATP synthases for oxidative phosphorylation by non-fermentative alkaliphiles. This creates a problem with respect to tenets of the chemiosmotic model that remains the core model for the bioenergetics of oxidative phosphorylation. Each of these facets of alkaliphilic bacteria will be discussed with a focus on extremely alkaliphilic *Bacillus* strains. These alkaliphilic bacteria have provided a cogent experimental system to probe adaptations that enable their growth and oxidative phosphorylation at high pH. Adaptations are clearly needed to enable secreted or partially exposed enzymes or protein complexes to function at the high external pH. Also, alkaliphiles must maintain a cytoplasmic pH that is significantly lower than the pH of the outside medium. This protects cytoplasmic components from an external pH that is alkaline enough to impair their stability or function. However, the pH gradient across the cytoplasmic membrane, with its orientation of more acidic inside than outside, is in the reverse of the productive orientation for bioenergetic work. The reversed gradient reduces the trans-membrane proton-motive force available to energize ATP synthesis. Multiple strategies are hypothesized to be involved in enabling alkaliphiles to circumvent the challenge of a low bulk proton-motive force energizing proton-coupled ATP synthesis at high pH.

## Introduction to Alkaliphilic Bacteria

The term alkaliphilic microorganisms or “alkaliphiles,” generally refers to microorganisms that grow well at pH values exceeding pH 9, often in the 10–13 range of pH (Horikoshi, 1999). Obligate alkaliphiles is a term used for alkaliphiles that grow only at pH values of ~pH 9 and above, while facultative alkaliphiles are strains that grow optimally under stringent alkaline conditions but are also capable of growing near neutral pH (Guffanti et al., 1986). Professor Koki Horikoshi, who has played a major role in developing interest in alkaliphilic bacteria and their capabilities, noted that only 16 papers on alkaliphilic bacteria had been published when he began his extensive studies of them in 1968 (Horikoshi, 1999). Since then, alkaliphiles have gained much more attention because of major contributions they make via the natural products they produce and the impact they have on diverse ecological settings. Interestingly, highly alkaliphilic bacteria, including some obligate alkaliphiles, have also been isolated from garden soils or other non-extremophilic settings. This suggests that these soils harbored niches that provided the necessary conditions for the persistence of such alkaliphiles (Horikoshi, 2006). Alkaliphilic bacteria are an important source of useful, stable enzymes and novel chemicals, including antimicrobials (Joshi et al., 2008; Fujinami and Fujisawa, 2010; Horikoshi, 2011; Sarethy et al., 2011; Ibrahim et al., 2012). Also, alkaliphile cells with specific capabilities can be used to carry out particular processes that benefit from the alkaline *milieu*, such as removing H_2_S from “sour” gas streams produced in the petrochemical industry (Sorokin et al., 2008). The ecological niches of alkaliphiles are remarkably diverse, e.g., ranging from alkaline soda lakes (Jones et al., 1998; Grant et al., 2004), the hind-gut of insects (Thongaram et al., 2003, 2005), to soils subjected to ammonification and human industrial processes that generate high pH (Jones et al., 1998). Other interesting environments are the alkaline hydrothermal vents, which have been proposed to recapitulate conditions that existed during formation of early life forms (Martin and Russell, 2007; Mulkidjanian et al., 2007; Martin et al., 2008; Lane and Martin, 2012; Herschy et al., 2014). The microbial activities impact the properties of their ecological niches and the niches impact the bacteria, as the evolutionary dance between the niche and the microbial population unfolds.

The next two sections will first amplify biotechnological contributions of alkaliphiles and the soda lakes in which many of those alkaliphiles are found (Jones et al., 1998; Grant, 2003; Sorokin et al., 2011, 2014), and then expand upon additional alkaline ecosystems that are “early earth analogs” (Suzuki et al., 2013). The extremophile enzyme that will then be highlighted in the final section is the F_1_F_o_-ATP synthase that is found in Gram-positive alkaliphilic *Bacillus* strains, such as *Bacillus pseudofirmus* OF4 (Janto et al., 2011) and *B. halodurans* C-125 (Takami et al., 2000). These alkaliphiles carry out oxidative phosphorylation in support of non-fermentative growth. The finding that such alkaliphilic aerobes use proton-coupled ATP synthases was a surprising finding, since the apparently low bulk proton-motive force (PMF) had led to the expectation that synthesis would be coupled to larger bulk sodium-ion gradients (Hicks and Krulwich, 1990; Krulwich, 1995). The use of protons at low bulk PMF has led to re-consideration of the idea that the PMF, which drives ATP synthesis, is the electrochemical gradient of protons between the bacterial cytoplasm and the bulk external *milieu* as envisioned in Peter Mitchell’s ground-breaking work (Mitchell, 1961). More localized models have gained ground (Williams, 1978; Slater, 1987; Heberle et al., 1994; Krulwich, 1995; Yumoto, 2002; Goto et al., 2005; Krulwich and Ito, 2013). As will be discussed, these models include fast movement of protons along the outer surface of the coupling membrane between proton pumps and ATP synthases. Other models include membrane properties that can increase proximity between pumps and synthases. A variety of emerging new findings now provide new opportunities to explore features that may together circumvent the “bioenergetic challenge” of proton-coupled ATP synthesis at low bulk PMF.

## Applications of Alkaliphilic Bacteria and Their Enzymes

A comprehensive review of established and proposed applications of alkaliphilic cells and alkaliphile enzymes to industrial uses is beyond the scope of this review; several such reviews have already been mentioned above. The aim of this section is, rather, to present a selection of applications in order to underscore the breadth of alkaliphile contributions for those whose interest in alkaliphiles are attuned mostly to the ecological or bioenergetic aspects of bacterial alkaliphily.

A major contribution of alkaliphiles to enzymes used in industry is the diversity of enzymes with activity optima shifted to the alkaline pH region. Mesophilic bacteria produce enzymes with similar activities but without enzymatic capacity at elevated pH. Alkaliphile enzymes from aerobic and anaerobic alkaliphilic bacteria tend, as expected, to have activity profiles that included higher pH values than displayed by mesophile enzymes. The added attraction was that they also often had additional capacities, e.g., some with high temperature optima and others with low temperature optima that increased the range of environments in which they were catalytically competent (Kumar and Takagi, 1999; Fujinami and Fujisawa, 2010; Horikoshi, 2011; Sarethy et al., 2011). Examples of alkaliphile enzymes and their uses include alkaline proteases, which are used as detergent additives and for removing hair from hides; starch-degrading amylases with elevated pH optima are also suitable for laundry use and debranching enzymes, together with amylase, play a role in stain removal (Ito et al., 1989; Gessesse et al., 2003; Sarethy et al., 2011); alkaline keratinases can degrade feathers that are often unwanted by-products of other processes (Kojima et al., 2006); and cyclomaltodextrin glucanotransferases (CGTases) from alkaliphilic strains enhance the production of cyclodextrins (CDs), which are used in pharmaceuticals, foodstuffs, and for chemical interactions (Horikoshi, 1999; Qi and Zimmermann, 2005; Fujinami and Fujisawa, 2010). Alkaliphiles also produce useful metabolites, including antibiotics. Among other metabolites, carotenoids are worth mentioning. They are for example responsible for the yellow color of many alkaliphilic *Bacillus* strains (Aono and Horikoshi, 1991).

In addition to useful alkaliphile enzymes and metabolites, there are many processes that can utilize these extremophiles, sometimes a mixture of alkaliphiles and other bacterial cells, to produce the desired changes. A classic example is the presence of *Alkalibacterium* strains as part of the microbiota involved in production of indigo blue dye from various plants in Europe, Japan, Korea, and elsewhere. Successive oxidation and reduction steps occur in a dye vat, in which the alkaliphile strains have important roles in the reducing phases (Aino et al., 2010; Park et al., 2012). Another example involving participation of alkaliphilic bacterial cells in a reduction process is the Fe(III)-reducing bacterium “*Alkaliphilus metalliredigens* QYMF” which could have useful applications to metal-contaminated alkaline environments (Ye et al., 2004; Roh et al., 2007). In another scenario, thermo-alkaliphilic *Bacillus* strains isolated from a textile wastewater drain were found to grow at pH 9.3–10 at 60–65°C (Paar et al., 2001). The most stable catalase among the strains was isolated from a strain designated *Bacillus* SF. This catalase was biochemically characterized and immobilized in order to recycle the remaining textile bleaching effluent without free enzyme contamination. A different situation is found when H_2_S is present in fuel gases and needs to be removed before combustion can proceed. Lithoautotrophs (sometimes called chemoautotrophs) can oxidize inorganic substrates such as H_2_S as an energy source. H_2_S-oxidizing bacteria of the genus *Thioalkalivibrio* were used in “lab-scale sulfide-removing bioreactors.” The results suggest that members of the *Thioalkalivibrio* genus have significant potential to oxidize sulfide under haloalkaline conditions in which desulfurization of natural gas is needed (Sorokin et al., 2008).

A final example of biotechnological deployment of alkaliphiles are microbial fuel cells (MFCs) (Logan et al., 2006). In the developing area of MFC devices, bacteria oxidize organic or inorganic substrates and generate current. As electrons are produced during the oxidation reactions, they are transferred to the anode, the negative terminal. They then flow to the cathode, the positive terminal, via conductive material whose properties support conditions for producing electricity. Among the bacteria that have been used for MFCs is a psychrophilic alkaliphile from seawater, *Pseudomonas alcaliphila* MBR (Yumoto et al., 2001), which releases phenazine-1-carboxylic acid under alkaline conditions (Zhang et al., 2011). A second, Gram-positive alkaliphile has been used to generate “bioelectricity” in an MFC. It is *Corynebacterium* sp. strain MFC03, which uses organic compounds, e.g., glucose, as electron donors at pH 9. The electron transfer mechanism which it employs appears to rely primarily on redox compounds that are excreted into the medium (Liu et al., 2010).

The examples given here underline the diversity of alkaliphile enzymes that have potential applications to industrial settings. Those applications continue to be identified with an increasing pace.

## Association of Alkaliphiles with Soda Lakes, Alkaline Hydrothermal Vents, and Other Serpentinizing Ecosystems

Many of the diverse alkaliphiles that display novel physiological features and potential applications were isolated from soda lakes found in various continents and settings (Borkar, 2015). Their properties, in addition to alkaliphily, cover wide tolerance ranges for salinity, temperature, and/or hydrogen levels. Soda lake alkaliphiles also participate in biogeochemical redox cycles of carbon, sulfur, and nitrogen (Sorokin et al., 2008, 2011, 2014), exhibiting capacities that are not found in alkaliphiles from other environments. Soda lake alkaliphiles are found extensively in parts of Asia (e.g., China and Russia) and in Africa (Jones et al., 1998; Sorokin et al., 2011), but there are other well-studied alkaliphiles that were found in places where soda lakes are less frequent, e.g., Lake Mono in California (Humayoun et al., 2003). As noted, the alkaliphiles isolated from soda lakes are often poly-extremophiles, e.g., *Natranaerobius thermophilus*, an anaerobe, which was isolated from the hypersaline lake Wadi El Natrun, Egypt and is resistant to high sodium-ion concentrations, pH, and temperature (Mesbah et al., 2009). The haloalkalithermophile *N. thermophilus* is an example of poly-extremophiles. In general, each of the individual resistances of a poly-extremophile is less extreme than the resistance observed for singly extremophilic bacteria, but the trade-off enables viability under multiple simultaneous stresses (Bowers et al, 2009; Mesbah et al., 2009; Mesbah and Wiegel, 2012).

Hydrothermal chimneys and black smoker vents that support biological communities were found in 1979 associated with mid-ocean ridges that have underlying layers of magma, whose cooling was a source of energy (Spiess et al., 1980). Subsequent findings of numerous vent fields were reported, followed by reports of the “Lost City Hydrothermal Field,” which was found at a different water depth and was shown to have novel properties. It is now recognized as a serpentinizing ecosystem (Kelley et al., 2005) with active carbonate structures and a broad range of springs, including some that have high temperatures and others that are in somewhat cooler settings. Their microbial cohort includes a dominant number of Archaea, and its bacterial cohort includes Gram-negative Epsilonproteobacteria and Gammaproteobacteria as well as Gram-positive Firmicutes that are relatives of known Firmicute sulfur- and methane-oxidizers (Schrenk et al., 2004). Brazelton et al. (2013) noted that the ultrabasic serpentinite springs can provide “windows” into the marine sub-surface, in an analogous way to the information obtained from deep hydrothermal vents. Recently, models of proton-based chemiosmosis were described that included a potential alkaline compartment for which there is a natural chemical origin. In turn, this model led to proposals that the alkaline hydrothermal vents could be a site for early development of cells (Martin and Russell, 2007; Martin et al., 2008; Lane et al., 2010; Lane and Martin, 2012). Others have characterized different ecological settings, which are terrestrial but anoxic geothermal fields; they proposed that these might be possible sites of the earliest life, based more on sodium-ion-coupling, which has been found in bacteria that have sodium pumps but are lower energy-consumers than bacteria that use proton-based oxidative phosphorylation, e.g., *Ilyobacter tartaricus* and *Propionigenium modestum* (Dimroth, 1997). Proton-coupling may have emerged early in alkaline hydrothermal vents, but may not have been immediately able to make levels of available energy comparable to levels made available by oxidative phosphorylation which involves electron transport by respiratory chain complexes and oxygen reduction, as it provides energy for proton-coupled ATP synthesis (Dimroth and Cook, 2004; Mulkidjanian et al., 2008, 2012). Questions regarding whether either Na^+^ or H^+^ coupling came “first” in evolutionary terms, or whether it is possible that they may have evolved in parallel in different niches, will not be addressed here. However, findings that indicate early presence of alkaliphilic bacteria are emerging from studies of specific serpentinizing ecosystems, and will be described.

**Figure 1:**
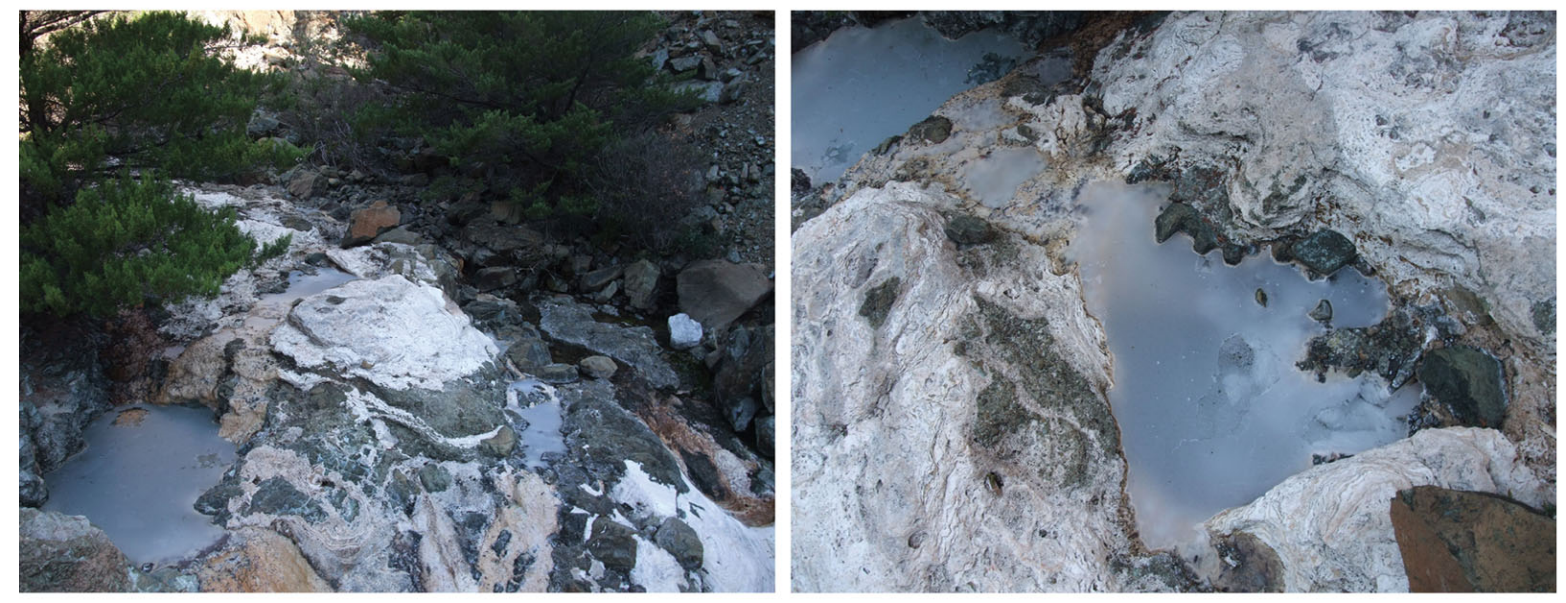
***Serpentimonas* strains have been isolated from the Barnes springs at The Cedars**. The images show Barnes spring complex (left) and Barns spring 5 (right). The Cedars is an area in northern California noted for the presence of abundant ultrabasic, highly alkaline, springs (pH 11–12) as the product of serpentinization. Serpentinization is a process involving reaction of water with ultramafic minerals, which have low silica content but are rich in minerals such as olivine. The interaction leads to formation of a new set of minerals including serpentine along with methane, hydrogen, and alkaline fluids. Barnes spring complex is one of the largest complexes in this site. The spring waters discharged from the sub-surface contain calcium as the result of serpentinization. The calcium reacts with carbonate at the surface of the spring, producing calcium carbonate precipitate that covers the surface of the springs (Frost and Beard, 2007; Suzuki et al., 2013).

Like The Lost City Hydrothermal Fields, which were noted earlier, The Cedars is an active serpentinization site, but it is located on the coast of northern California and is fed by deep groundwater (Suzuki et al., 2013) (Figure 1). The Cedars comprises highly basic and reducing springs with low salinity and has bacteria from various phyla, including clostridia and Betaproteobacteria. A highly alkaliphilic, hydrogen-oxidizing Gram-negative Betaproteobacterium was isolated from a spring with a pH of 11.6. It displays a pH optimum for growth of pH 11 and it exhibits autotrophic growth on hydrogen, calcium carbonate, and oxygen. The name “*Serpentinomonas raichei* A1” has been proposed for this extremely alkaliphilic organism and its gene sequence indicates that it has an ATP synthase that couples to protons rather than sodium ions (Suzuki et al., 2014). As discussed in the final section of this review, *S. raichei* A1 and several other Gram-negative alkaliphiles that couple ATP synthesis to protons differ from Gram-positive alkaliphiles in the amino acid sequence motifs of two key subunits of the ATP synthase. This difference may provide clues to the range of adaptations that enable proton-coupled ATP synthesis to proceed under the adverse conditions of a low “bulk” PMF. More interesting observations may arise from an increasing number of alkaline ground water (Roadcap et al., 2006) and serpentinite sites whose bacterial cohort can be studied (Crespo-Medina et al., 2014; Meyer-Dombard et al., 2015).

## The Bioenergetic Challenge Imposed upon Alkaliphiles That Carry Out Proton-Coupled Oxidative Phosphorylation

### A schematic profile of bioenergetic cycles and related cell structures of extremely alkaliphilic *Bacillus* strains

**Figure 2:**
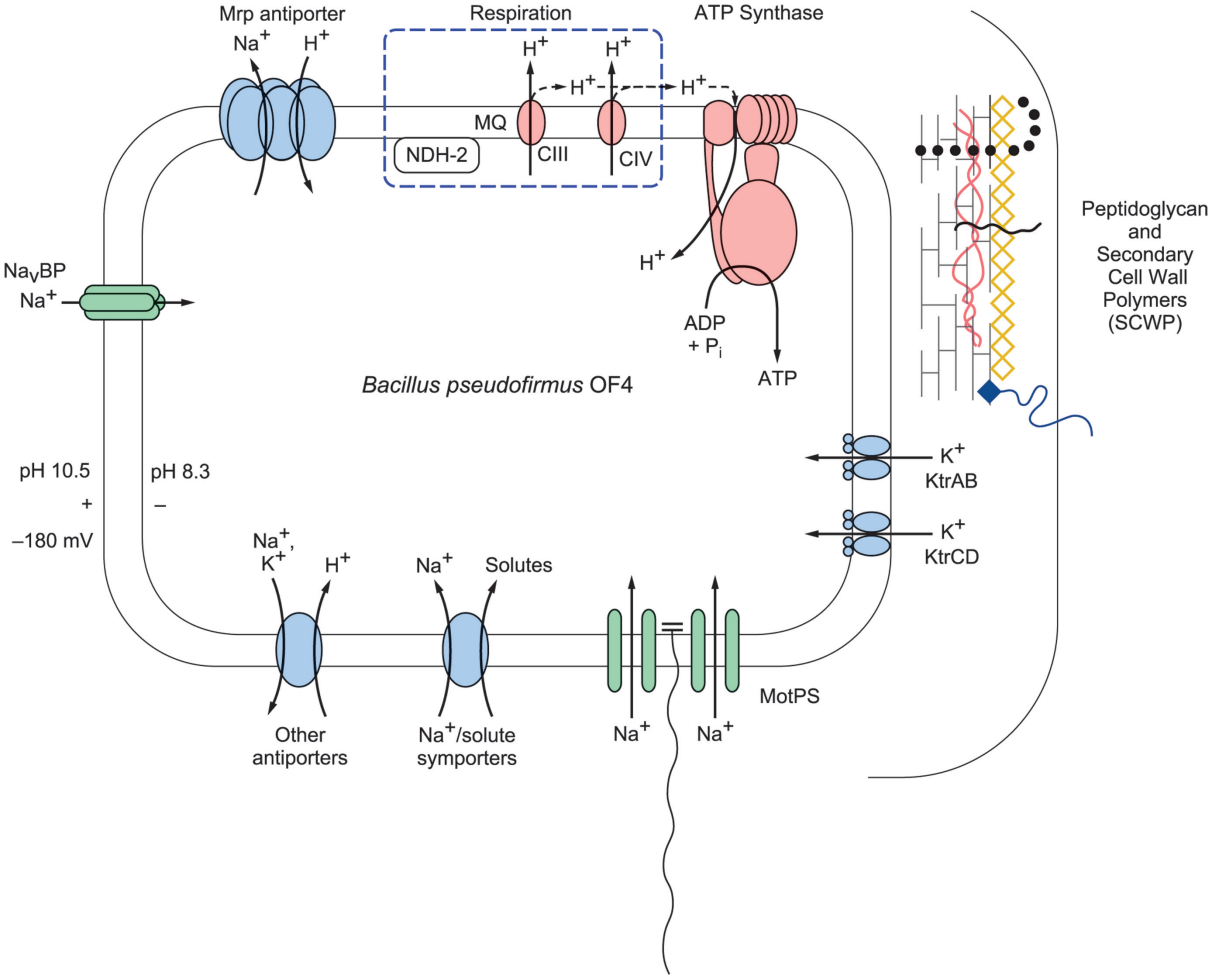
**A schematic representation of selected bioenergetic and cell surface properties of alkaliphilic *Bacillus pseudofirmus* OF4**. Electron transfer is initiated by dehydrogenases such as NADH dehydrogenase (designated NDH-2), a non-proton-pumping enzyme that replaces complex I in many bacteria. The dehydrogenases donate electrons to the menaquinone (MQ) pool in the membrane. From reduced MQ, the electrons move on through the two proton-pumping complexes, the menaquinol:cytochrome *c* oxidoreductase (complex III, labeled CIII in the figure) and cytochrome *c* oxidase (complex IV, labeled CIV in the figure), which pump protons out of the cell into the bulk medium as molecular oxygen is reduced. These protons are shown with the solid line. Evidence has been presented for retention of other protons on or near the membrane where they move laterally and may reach PMF users such as ATP synthase before equilibrating with the bulk medium outside the cell (Heberle et al., 1994; Sandén et al., 2010; Rieger et al., 2014). These protons are shown with a dashed line. The bioenergetic impact and possible mechanisms of that delay of proton equilibration will be discussed in the final section of this review. In addition to energizing ATP synthesis, the proton-motive force (PMF) generated by respiration energizes the cation/proton antiporters that catalyze electrogenic import of more protons than the number of sodium ions that are exported in the antiporter turnover. The inward movement of protons contributes to the maintenance of a low pH inside the alkaliphile cytoplasm relative to the pH in the medium. This facilitates maintenance of a cytoplasmic pH of 8.3 while the external pH is 10.5, an “inverted” ΔpH. Additional sodium ions enter through the MotPS sodium-ion channels (Ito et al., 2004a) that power motility and through a voltage-gated sodium-ion channel, Na_v_BP (Ito et al., 2004b); the influxes of sodium ions complete a sodium-ion cycle, enabling continued uptake of protons via antiporters. Ktr potassium ion importers similarly contribute to completion of a potassium ion cycle. The net chemiosmotically productive, trans-membrane potential (ΔΨ) is substantial, so that the total of the inverted ΔpH and the ΔΨ yields a low but productively oriented bulk PMF.

The central challenge for extremely alkaliphilic bacteria is the maintenance of a cytoplasmic pH that is significantly lower than the highly alkaline external *milieu* in which they grow. As depicted in Figure 2, *B. pseudofirmus* OF4 grows in media with a pH of 10.5. Under these conditions, alkaliphilic *Bacillus* strains use antiporters to catalyze an electrogenic exchange of outwardly moving sodium ions, or in some instances potassium ions, for a greater number of entering protons. The multi-subunit Mrp-type of antiporter comprises 7 hydrophobic gene products, or a 6-subunit version in which MrpA and MrpB are fused. This membrane-embedded antiporter plays an essential role in catalyzing the electrogenic antiport in support of alkaliphily in intensively studied organisms such as *B. pseudofirmus* OF4 (Janto et al., 2011) and *B. halodurans* C-125 (Takami et al., 2000) and *B. alcalophilus*, which appears to have a potassium ion cycle that includes at least one of its two Mrp antiporters (Attie et al., 2014). A point mutation that rendered *B. halodurans* C-125 non-alkaliphilic led to characterization of the Mrp-type antiporters (Hamamoto et al., 1994), which are also found in both Gram-positive and Gram-negative non-alkaliphiles. Among the bacteria in which Mrp antiporters have been found to have important roles are non-alkaliphiles that are able to grow under moderately alkaline conditions, e.g., *B. subtilis*, *P. aeruginosa*, *Rhizobium meliloti*, *Vibrio cholerae*, and *Staphylococcus aureus* (Hiramatsu et al., 1998; Putnoky et al., 1998; Ito et al., 1999; Kosono et al., 1999, 2005; Swartz et al., 2005; Dzioba-Winogrodzki et al., 2009). In addition to one or more Mrp cation/proton antiporters, alkaliphilic bacteria generally have a robust cohort of additional antiporters encoded by single genes (Takami et al., 2000; Janto et al., 2011).

It is crucial for maintenance of pH homeostasis in extreme alkaliphiles that there is continuous availability of the efflux cation so that the cation/proton antiporters have a substrate, be it sodium- or potassium-ions, to exchange with cell-external protons (Krulwich et al., 1985). The cation/proton antiporters are indispensable in enabling alkaliphiles to sustain a cytoplasmic pH that is often more than two pH units below the pH of the medium (Krulwich, 1995). For example, *B. pseudofirmus* OF4 maintains a cytoplasmic pH of ≤8.3 at an external pH of 10.8 and has an upper pH limit for growth at about pH 11.4 (Sturr et al., 1994). In addition to the cation/proton antiporters, several other paths for cation entry play roles in ensuring that sufficient cytoplasmic substrate for the antiporters is present at high pH. A major additional route of cation uptake is provided by the numerous solute uptake pathways that take up solutes together with sodium ions, which is energetically favorable because of the inwardly directed sodium-ion gradient (Figure 2). Also shown in Figure 2 are two additional entry routes for cations that contribute to pH homeostasis in alkaliphiles under high pH conditions. One route is provided by the cation uptake channels that power flagellar rotation, e.g., the MotPS sodium-ion channel in extremely alkaliphilic *Bacillus* strains including *B. halodurans* C-125 and *B. pseudofirmus* OF4 (Fujinami et al., 2009). Interestingly, some neutrophilic *Bacillus* strains, including *B. subtilis*, have a proton-conducting MotAB motility channel as well as a sodium-ion-conducting MotPS type (Ito et al., 2004a). A second group of channels that support cation uptake in alkaliphilic *Bacillus* strains are voltage-gated cation channels (Figure 2). A voltage-gated sodium-ion channel from *B. halodurans* C-125 was first noted by Durell and Guy (2001) and hypothesized to be a calcium ion channel. When subsequently cloned and characterized, it was found to be a voltage-gated sodium-ion channel and was designated NaChBac (Ren et al., 2001). Structural insights followed from studies of related proteins from a marine Alphaproteobacterium (Zhang et al., 2012) and from *Arcobacter butzleri* (Payandeh et al., 2012). Studies of the NaChBac homolog from alkaliphilic *B. pseudofirmus* OF4, Na_v_BP, revealed a sodium-ion-specific channel that is potentiated at high pH and has a role in chemotaxis as well as in cytoplasmic pH homeostasis (Ito et al., 2004b). By contrast, the *B. alcalophilus* member of the voltage-gated channel family has been shown to be a voltage-gated channel that is remarkably non-specific with respect to multiple monovalent and divalent cation substrates (DeCaen et al., 2014). This characteristic is consistent with the hypothesis that *B. alcalophilus* transporters can couple solute to diverse cation gradients (Attie et al., 2014).

When the pH of the outside medium of extreme alkaliphiles is raised to very high alkaline levels, the capacity to maintain a steady cytoplasmic pH below pH 8.5 is lost and an upward creep in the cytoplasmic pH is observed. For example, at the high end of the growth range for alkaliphilic *B. pseudofirmus* OF4, e.g., ≥11.2, the cytoplasmic pH rises to ~9.5 (Sturr et al., 1994). The basis for resistance to this unusually high cytoplasmic pH is not yet fully understood and is an intriguing part of alkaliphiles’ extremophilic capacity (Janto et al., 2011; Krulwich et al., 2011).

Another major problem is raised by the substantial “reversed” proton gradient (i.e., a significantly more acidic cytoplasm than the pH of the medium). This orientation of the pH gradient reduces the total PMF that drives proton-coupled processes such as proton-coupled ATP synthesis, and in many bacteria, proton-coupled uptake of solutes as well as motility. The productive orientation of the PMF, which is an electrochemical gradient, consists of an inwardly directed chemical gradient of protons (ΔpH) and a trans-membrane potential oriented with the positive side on the outside (ΔΨ) across the bacterial membrane. While the reversal of the ΔpH is accompanied by a rise in the ΔΨ, the increment does not fully compensate for the reversal of the ΔpH (Krulwich, 1995). Therefore, the net PMF available for bioenergetic work is significantly reduced. How then, do aerobic alkaliphiles carry out robust PMF-dependent work? The expected answer was that the low inwardly directed PMF would not be used by alkaliphiles but that the larger sodium-ion gradient could energize ATP synthesis just as it energizes alkaliphiles’ solute uptake systems and motility (Figure 2). As mentioned earlier, there are indeed F_1_F_o_-ATP synthases that are coupled to sodium ions. However, these synthases are found in bacteria such as *I. tartaricus* and *P. modestum* but not in bacteria that derive energy from electron transport during oxidative phosphorylation, such as aerobic alkaliphiles (Dimroth and Cook, 2004). *B. pseudofirmus* OF4, which was originally named *B. firmus* OF4, was the first alkaliphile found to use a proton-coupled ATP synthase, in experiments using purified synthase reconstituted and assayed in proteoliposomes (Hicks and Krulwich, 1990). It was also noted that there was barely detectable hydrolytic activity, a typical feature of alkaliphile ATP synthases. Without suppression of ATP hydrolysis by alkaliphile synthases, alkaliphiles would be susceptible to loss of ATP via hydrolysis when the low PMF dipped stochastically. A study of the molar growth yields of *B. pseudofirmus* OF4 was conducted in a continuous culture that was held at a range of specific pH values between pH 7.5 and 11.2, with vigorous aeration. The results showed that *B. pseudofirmus* OF4 grew throughout that range, with higher molar growth yields at pH 10.5 than at pH 7.5 (Sturr et al., 1994). Subsequently, the expected use of protons by *B. alcalophilus* was reported (Hoffmann and Dimroth, 1991), and, since then, other aerobic and alkaliphilic strains have similarly exhibited proton-coupling, e.g., thermo-alkaliphilic *Caldalkalibacillus thermarum* strain TA2.A1, which only exhibits non-fermentative growth at alkaline pH values and couples the ATP synthase to protons (Cook et al., 2003; McMillan et al., 2009).

### Alkaliphile F_1_F_o_-ATP synthases and an overview of adaptations that support their function at low PMF

**Figure 3:**
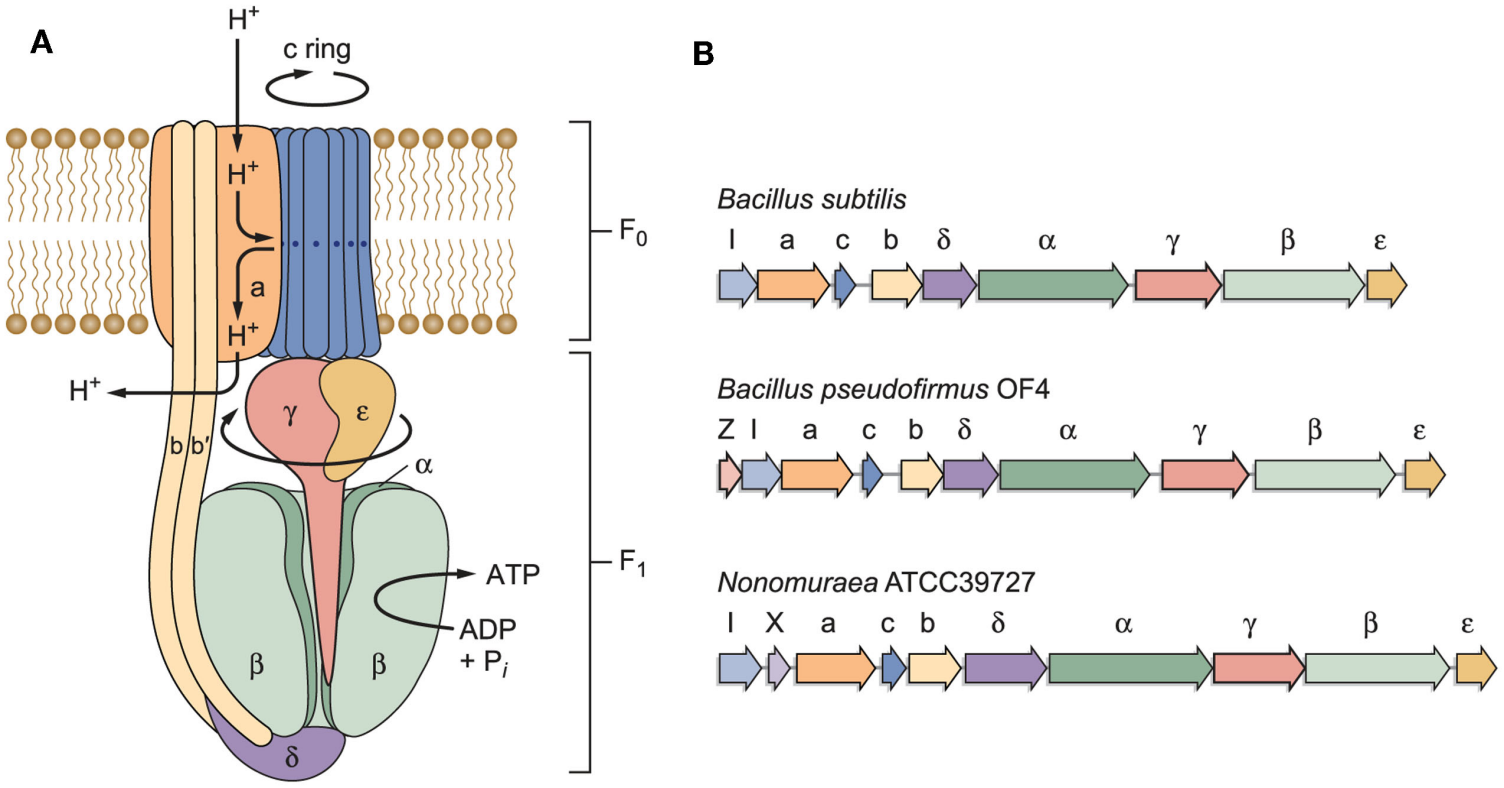
**(A)** A model of a bacterial F_1_F_o_-ATP synthase and **(B)** schematic examples of bacterial *atp* operons. The F_1_ sector containing the α/β catalytic sites is bound by stator elements to the membrane-embedded F_o_ portion of the complex. The F_o_ sector contains the proton-conducting elements utilized to transform proton electrochemical gradients into rotational energy that supports ATP synthesis. Of special interest here are the *a*- and *c*-subunits in the F_o_ that work together to convert the proton electrochemical gradient into *c*-ring rotation, ultimately leading to ATP synthesis. Non-fermentative alkaliphilic *Bacillus* strains have adaptations in both subunits that are hypothesized to help capture protons and to facilitate the binding of those protons to the essential carboxylate in the *c*-subunit (E54 in *B. pseudofirmus* OF4). Pathway(s) for proton uptake and exit are hypothesized to be comprised of two half-channels in the *a*-subunit (Angevine and Fillingame, 2003). One half-channel is an uptake channel that delivers protons from the outside down their electrochemical gradient to the essential carboxylate in the *c*-subunit. The other half-channel is an exit pathway, ultimately releasing protons in the cytoplasm. As detailed further in the text, roles have been hypothesized and in some instances assigned to the “alkaliphile-specific motifs” that are found in the trans-membrane helices (TMH) of the *c-*subunit, in TMH1: AxAxAVA and in TMH2: PxxExxP. **(B)** Schematic examples of bacterial *atp* operons. The *atp* operon of *B. subtilis* is typical of many *atp* operons from neutralophilic bacteria, with one gene, *atpI*, just upstream of the eight structural genes of the enzyme complex. Another type is found in a variety of Gram-positive species, especially *Bacillus* species. The *atp* operon of alkaliphilic *B. pseudofirmus* OF4 is similar to the *B. subtilis* example, but has a small additional gene just upstream of *atpI*, designated *atpZ*. This gene encodes a hydrophobic protein that has a role in Mg^2+^ homeostasis (Hicks et al., 2003). An example of a non-alkaliphile *atp* operon, from actinomycete *Nonomuraea* sp. ATCC 39727, also includes a gene predicted to encode a hydrophobic protein, *orfX*, which is found between *atpI* and the gene encoding the *a*-subunit (Gaballo et al., 2006).

The model of an F_1_F_o_-ATP synthase in Figure 3A is based on work by other investigators who have developed key features of this rotary machine (Stock et al., 2000; Yoshida et al., 2001; Boyer, 2002; Walker, 2013). The model indicates a path of protons through a “half-channel” of the membrane-embedded *a-*subunit, which leads to an interface that enables the proton to move from the *a-*subunit to the *c-*rotor ring, and subsequently be released through another half-channel into the bacterial cytoplasm after it has completed a full rotation (Angevine and Fillingame, 2003; Dong and Fillingame, 2010). Recently, a structural study of the *a-*subunit from the algae *Polytomella* sp., which has a dimeric F-type ATP synthase structure, has shown that the membrane-embedded helices of the *a-*subunit stator are horizontal relative to the *c-*ring (Allegretti et al., 2015). When the sequences of the proton-conducting *a-* and *c-*subunits of both *B. pseudofirmus* OF4 and *B. alcalophilus* were first obtained, it was evident that there were sequence deviations from neutralophilic bacterial homologs (Ivey and Krulwich, 1991, 1992). It was anticipated that these “alkaliphile motifs” would turn out to be adaptive to the challenges of the alkaliphile setting. In addition to the major problem of an insufficient PMF to account for the ATP synthesis observed, challenges that were likely to require special adaptations included the risk of proton loss to an alkaline environment before the protons reach a proton binding site on the rotor ring or while the rotor ring is in rotation within the lipid *milieu*. In Figure 3B, the operons encoding three different F_1_F_o_-ATP synthases, from *B. subtilis*, alkaliphilic *B. pseudofirmus* OF4, and an actinomycete example, *Nonomuraea* ATCC39727 are compared. Like neutralophilic *B. subtilis*, alkaliphilic strain *B. pseudofirmus* OF4, has an *atp* operon with the expected eight structural, ATP synthase protein-encoding genes and an *atpI* gene whose protein product contributes to stability and assembly of the synthase (Suzuki et al., 2007; Liu et al., 2013). However, the *B. pseudofirmus* OF4 operon has an additional gene, designated *atpZ*, that is operon-associated but is absent in *B. subtilis* (Figure 3B). Some other *atp* operons have an additional gene, as shown for the actinomycete *Nonomuraea* which also has a ninth gene (designated as *orfX*) as does *B. pseudofirmus* OF4 (Figure 3B). But the predicted product of *Nonomuraea orfX* is different in sequence, size, and location relative to the alkaliphile AtpZ (Liu et al., 2013). In *B. pseudofirmus* OF4, the *atpZ* gene has been shown to enhance the ability of the alkaliphile to acquire sufficient magnesium, which is challenging at elevated pH (Hicks et al., 2003). Lee et al. (2014) suggested that *atpZ* might have an evolutionary link to mitochondrial calcium uniporters (MCUs) (De Stefani et al., 2011).

### Adaptations of the *a-*subunit in support of ATP synthesis in alkaliphilic bacteria

**Figure 4:**
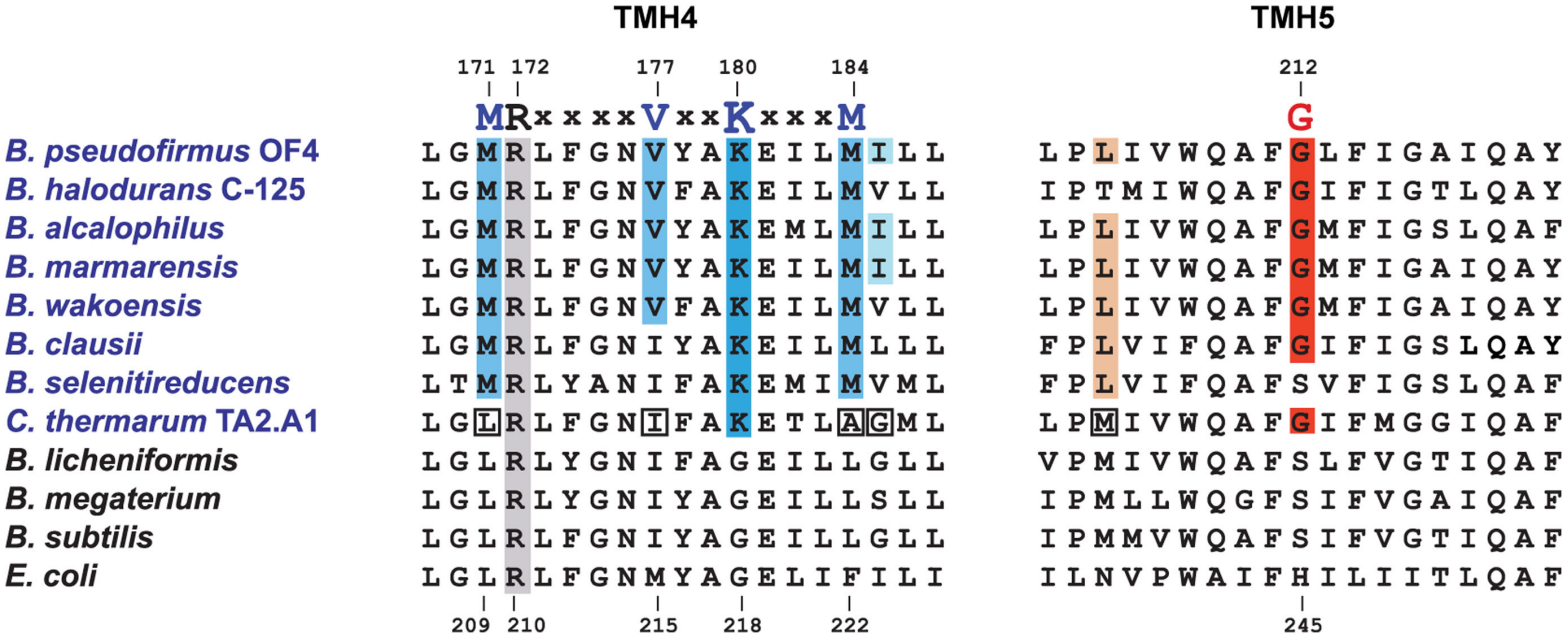
**Special features of alkaliphilic *Bacillus* ATP synthase *a*-subunits**. Portions of trans-membrane helices (TMH) 4 and 5 contain many of the residues that form the putative aqueous channels through which protons are delivered to a deprotonated, key carboxylate in the *c*-ring and subsequently exit from the *c*-ring to the cytoplasm. Alkaliphilic species are in blue. The essential arginine that is conserved in all *a*-subunits is highlighted in gray (position 172 in *B. pseudofirmus* OF4 and 210 in *E. coli*). In TMH4, alkaliphiles have a generally conserved motif of M(R)xxxxVxxKxxxM shown in blue, with the critical lysine in a darker blue. V177 and K180 are positioned to be part of the putative proton uptake pathway from the periplasm in analogy to the equivalent *E. coli* residues (Dong and Fillingame, 2010). The glycine at position 212 in *B. pseudofirmus* OF4 (in red) is conserved in alkaliphiles and is likely to be a key residue in the uptake pathway. A few alkaliphiles lack V177 or G212 and are hypothesized to be more moderately alkaliphilic. This is the case here for the poly-extremophilic *C. thermarum* TA2.A1, which is the only alkaliphile in a group of 21 *a*-subunits (not shown) that lacks M171 and M184, as well as the generally conserved V177. The *C. thermarum* TA2.A1 residues that deviate from the alkaliphile consensus are in white boxes. The isoleucine at 185 (pale blue) and the leucine at 205 (brown) are less conserved than the other relevant residues. See Table 1 for a summary of the conservation of the alkaliphile-specific residues and Table 2 for the phenotypes of relevant mutants.

Aligned amino acid sequences of the *a-*subunits from several alkaliphilic *Bacillus* species are shown in Figure 4 along with those from thermo-alkaliphilic *C. thermarum* TA2.A1, three non-alkaliphilic *Bacillus* strains, and *Escherichia coli*. When the alkaliphiles were first studied, there were enough sequences available to unequivocally identify K180 of trans-membrane helix-4 (TMH4) as a residue of interest and G212 of TMH5 as a possible interacting partner for K180. Their hypothesized function was to act as an important impediment to proton loss from the entry half-channel of the *a-*subunit and thus promote successful movement of protons to the ion-binding sites of the *c-*ring. In the earliest mutagenesis study, the K180 residue was replaced by glycine, the consensus residue for non-alkaliphiles, resulting in major loss of growth on malate at pH 10.5 but not 7.5 and major loss of ATP synthesis by ADP + P_i_-loaded membrane vesicles while replacement of G212 by serine had a much smaller defect (Wang et al., 2004). In a later study, K180 was replaced by alanine, glycine, cysteine, arginine, and histidine. The resulting ATP synthases were all defective in their ability to grow on malate and carry out ATP synthesis. It was also noted that the alanine-, glycine-, and histidine-containing mutants were considerably more sensitive to the uncoupler carbonyl cyanide *m*-chlorophenyl hydrazone (CCCP) than the WT strain (Fujisawa et al., 2010). The synthase in which K180 was changed to arginine exhibited no synthesis under any tested conditions, although the same substitution in *C. thermarum* TA2.A1 was functional (McMillan et al., 2007).

At the time of this initial work, it was noted that additional motifs were probably present in the different *a-*subunits. The larger data base of alkaliphile sequences now confirms that the motifs associated with the alkaliphile *a-*subunits are more comprehensive. Again, the alkaliphile motifs are distinct from those of neutralophilic *Bacillus* strains as well as *C. thermarum* TA2.A1, and some of the *Bacillus* alkaliphiles that are somewhat less alkaliphilic than the top five strains in the alignment (Figure 4; Table 1). Subsequently, when more sequences became available, multiple alkaliphile sequences revealed other residues in membrane segments that were hypothesized to be in or very close to the *a-*subunit proton pathway. These were M171, V177, M184, I185, and L205 (Fujisawa et al., 2010) (Table 1). Except for *C. thermarum* TA2.A1, the two methionines are conserved in all *Bacillus* alkaliphiles, and their mutation to the sequence found in neutralophiles resulted in impaired growth on malate (Table 2). The mutation of V177 to the neutralophile consensus also resulted in a significant loss of growth on malate, as did the mutations of the two other flagged residues of potential interest, I185 and I205 (Tables 1 and 2). No clear notion of the basis for their functional importance has yet emerged but as soon as more structural data become available in the future, a more detailed hypothesis will likely become feasible.

**Table 1:** **The sequence similarity among 21 *Bacillus* alkaliphiles in identified alkaliphile amino acid variants in the ATP synthase *a*-subunit**.

*Bacillus pseudofirmus* OF4 residue	M171	V177	K180	M184	I185	L205	G212
*Bacillus* alkaliphile conservation	20/21	17/21	21/21	20/21	9/21	9/21	17/21
*Bacillus* neutralophile consensus	L	I	G	L	G (S/T)	M (T/F)	S
*C. thermarum* TA2.A1 residue	L	I	K^a^	A	G	M	G^a^

*^a^This residue matches the alkaliphile consensus. The other residues of C. thermarum TA2.A1 deviate from the alkaliphile consensus (and, for the most part, correspond to the *Bacillus* neutralophile consensus)*.

**Table 2:** **Phenotypes of *B. pseudofirmus* OF4 ATP synthase *a*-subunit mutants**.

Mutation	Growth at pH 10.5 with malate (% of WT)	Growth at pH 7.5 with malate (% of WT)
M171L	49	78
V177I	32	55
K180G	18	86
M184L	20	30
I185G	49	84
G212S	100	86
L205M	30	33

*Data are from Wang et al. (2004) and Fujisawa et al. (2010). Growth was for 16 h. The mutations were based on the equivalent consensus *Bacillus* neutralophile *a*-subunit residues. Standard deviations can be found in those references. Although the G212S mutant did not show a defect when grown in malate-replete media, the mutant exhibited defects when it was starved extensively and reenergized with malate compared to the wild-type. Whereas the WT after 1 h of re-energization showed an increased ΔGp/Δp and lowered cytoplasmic pH, the G212S mutant exhibited the opposite pattern, with a *decreased* ΔGp/Δp and an *increased* cytoplasmic pH from time 0 (Wang et al., 2004). The ΔGp is the phosphorylation potential ($\Delta G_{0}^{’}$ + RT ln[ADP][Pi]/[ATP]) and the Δp is the proton-motive force (arithmetic sum of ΔpH in mV and the membrane potential)*.

### Adaptations of the *c-*subunit that support ATP synthesis by alkaliphilic bacteria

#### Two Major Motifs of the *c-*Subunit of Alkaliphiles

**Figure 5:**
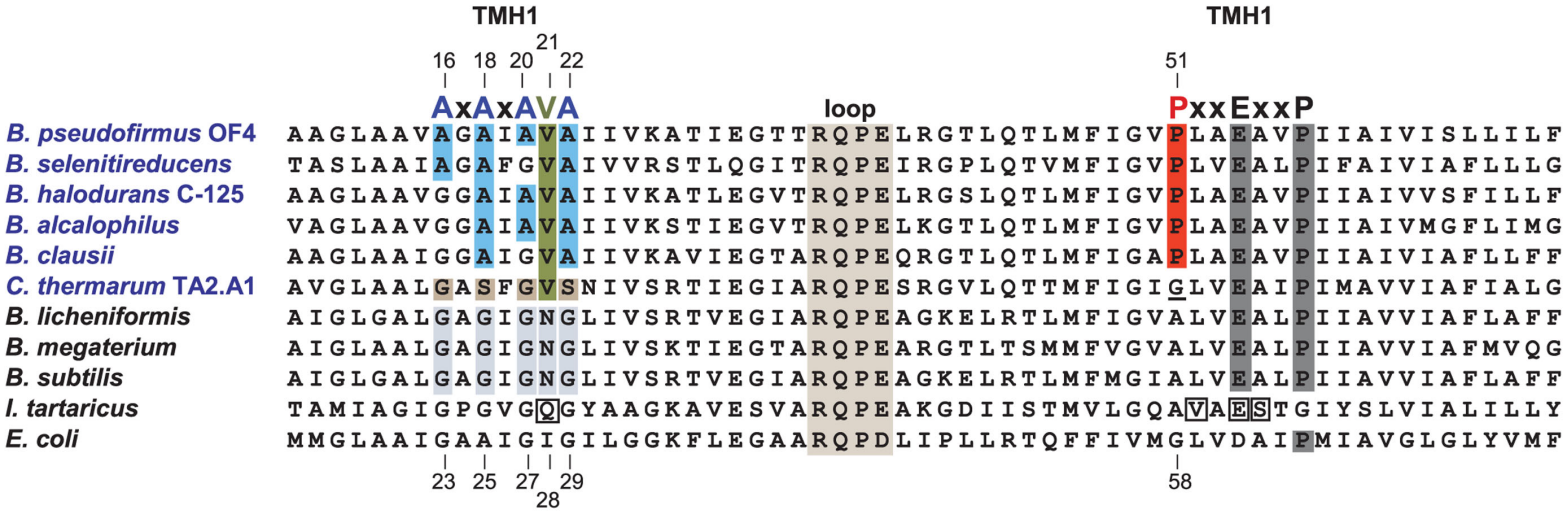
Alignment of the Bacillus alkaliphiles c-subunits with those from Bacillus neutralophiles, the Na+-binding c-subunit of I. tartarticus and the ***c***-subunit from E. coli. Neutralophilic *Bacillus* species have a well-conserved TMH1 motif of GxGxGNG, which is shaded in gray. *Bacillus* alkaliphiles names are in blue. The TMH1 motif of these alkaliphiles, shown in blue, is AxAxA(V)A. The valine (V21) is highlighted in green. A different deviation from the non-alkaliphilic bacteria TMH1 motif, is the GxSxGVS sequence found in the poly-extremophilic, thermo-alkaliphilic *C. thermarum* TA2.A1, as highlighted in brown. The second TMH2 alkaliphile motif is PxxExxP, which includes the unique alkaliphile proline (P51 in *B. pseudofirmus* OF4) shown in red. P51 is indirectly required for water coordination within the active site and may play a role in how protons are moved to the cytoplasm after complete *c*-ring rotation, in analogy to the proposed function of the equivalent *E. coli* residue, G58 (Steed and Fillingame, 2009). *C. thermarum* TA2.A1 is the only *Bacillus* alkaliphile among a group of 19 aligned alkaliphilic *Bacillus c-*subunits (not shown) that lacks this proline, instead containing a glycine (which is underlined) at this position. The direct Na^+^-coordinating residues of the *I. tartarticusc*-subunit are boxed (Meier et al., 2005, 2009); these are Q32, V63, E65, and S66. The numbering at top is from *B. pseudofirmus* OF4 while the bottom numbering is from *E. coli*. To improve the clarity of the figure, a number of residues at the N-terminus and C-terminus of the different *c*-subunits were omitted. Mutant strains were constructed based on the consensus *Bacillus* neutralophile residues. The relevant phenotypes of mutant strains are as follows. In the AXAXA(V)A motif, single alanine to glycine mutants are reduced in pH 10.5 malate growth approximately 30–40% compared to WT (Liu et al., 2009, 2011). The double mutants A18G/A22G and A20G/A22G were modestly impaired in high pH malate growth compared to the single A18G, A20G, or A22G mutants. In contrast, the double mutants that included an A16G mutation were significantly reduced in malate-dependent growth at high pH, with the A16G/A18G mutant exhibiting just 36% of WT growth while the growth of the A16G/A20G mutant was only 9% of WT growth. The ATP synthase from the A16G mutant in the *c-*subunit displayed *c*-rings with both a *c*13 (WT) and *c*12 stoichiometry while double A16G/A20G mutants displayed *c-*rings with only a *c*12 stoichiometry (Liu et al., 2010). The V21N mutant grew at similar rates to the WT under all tested conditions except at pH 10.5 with malate, where its doubling time, 3.7 h, was 2.5 times slower than the WT (Preiss et al., 2014). The proline mutant, P51A, exhibited a small reduction in malate growth at pH 7.5, which was 75% of WT, but was very sensitive to malate growth at pH 10.5, only 23% of WT (Liu et al., 2009).

In comparison with neutralophiles, alkaliphilic *Bacillus* strains have two major motifs in their *c-*subunit amino acid sequences. First, and located on the N-terminal helix of the hairpin like *c-*subunit, there is a 16AxAxAVA22 motif (using *B. pseudofirmus* OF4 numbering), which replaces the GxGxGNG motif found in neutralophilic *Bacillus* strains (Figure 5). However, only *B. pseudofirmus* OF4 has the full set of four alanines in the motif. Second, located on the C-terminal helix, a proline at position 51 appears. This proline is an alkaliphile-specific residue that is three residues in front of the critical, ion-binding glutamic acid residue, E54. In some cases, a second proline residue appears equidistantly positioned after E54. Together they form the motif 51PxxExxP57 (Arechaga and Jones, 2001). Mutagenesis studies on the two motifs showed that a quadruple mutant in which the four alanines of the AxAxAVA motif were changed to glycines resulted in loss of only 50% of the WT hydrolytic activity but loss of over 80% of the enzyme’s ATP synthesis capacity (Liu et al., 2009). A mutation of P51 to alanine led to a very significant loss in the ability to grow non-fermentatively at pH 10.5, while replacement of P51 with glycine led to mutants with growth deficits and proton leakiness (Liu et al., 2009). A mutation of the E54 residue, which is expected to have a central role in the ion-binding site, to D54, led to acute proton leakiness accompanied by almost total loss of the ability to grow on malate (Liu et al., 2009).

#### The Stoichiometry of the *c-*Rotor Ring and Impact of the Motifs

The stoichiometry of the *c-*rotor ring as well as the basis for the apparent impact of the *c-*subunit motifs were clarified when the crystal structure of the purified *c-*subunit rotor ring from alkaliphilic *B. pseudofirmus* OF4 was solved at 2.5 Å (Preiss et al., 2010). The stoichiometry of *c-*rotor rings from various organisms, i.e., the number of *c-*subunits in each ring, is in a range from 8 subunits in vertebrates to 13–15 in various photosynthetic organisms (Watt et al., 2010; Meier et al., 2011). Alkaliphilic bacteria such as *B. pseudofirmus* OF4 and thermoalkaliphilic *C. thermarum* TA2.A1 both have 13-subunits, near the upper end of the observed range (Meier et al., 2007; Matthies et al., 2009; Preiss et al., 2010). The *c*-rings from each bacterial species, plant, or animal, have a constant *c-*subunit stoichiometry, with each *c-*subunit coordinating one proton (or sodium ion, where applicable) via the glutamate or aspartate in each of the ion-binding sites. During the 360° rotation of the rotor ring, with the attached central stalk γϵ subunits, three ATPs are synthesized in the F_1_ sector β subunits. The number of coupling ions translocated for synthesis of the three molecules of ATP is determined by the *c*-ring stoichiometry: a larger *c-*ring is beneficial under circumstances of a lower PMF and vice versa; this notion is consistent with results of engineering of rotor ring stoichiometries both in upward and downward directions (Pogoryelov et al., 2012; Preiss et al., 2013).

**Figure 6:**
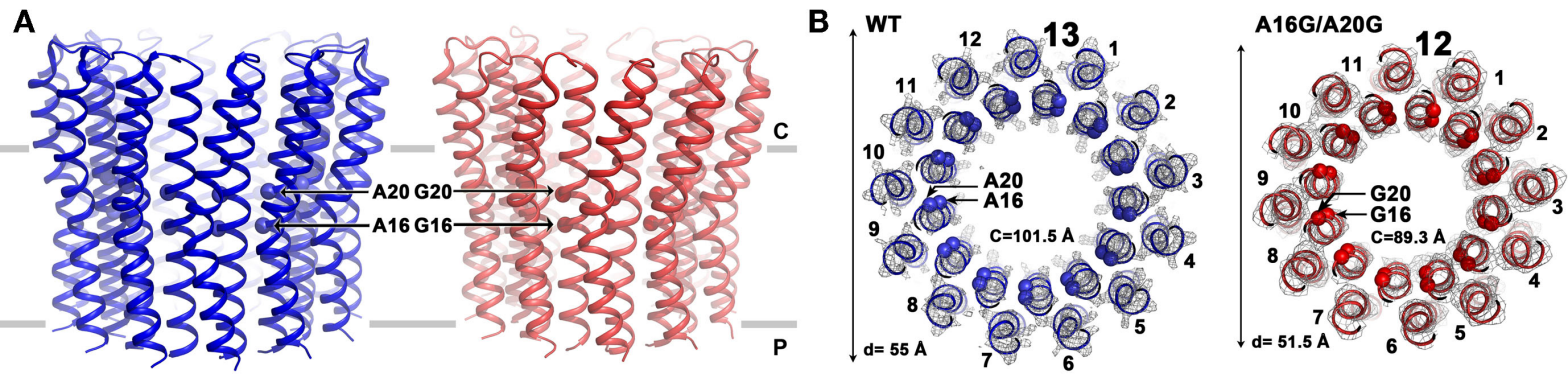
**Comparison of the structures of the *B. pseudofirmus* OF4 wild-type (WT) and the A16G/A20G mutant *c*-ring**. Side views **(A)** and top views **(B)** are shown for the WT and the double A16G/A20G mutant *c*-rings at the level of residue A16 and G16. Alanine and glycine residues 16 and 20 are located in the inner N-terminal helix at the middle region of the membrane. A mutation of A16G/A20G alters the *c*-ring stoichiometry from 13 (WT, blue, pdb code: 2 × 2v) to 12 (A16G/A20G mutant, red, pdb code: 3zo6) as visualized by atomic force microscopy and X-ray crystallography (Preiss et al., 2013). The mutations further reduced *c-to-c-*subunit distances at the level of A16 and A20, resulting in a smaller ring circumference (*C*) of only 89.3 Å (on level of G16) and a ring diameter of 51.5 Å. Helices are shown in cartoon representation, Cα of WT with both A16 and A20 shown as blue spheres and the double mutant with G16 and G20 shown as red spheres. Electron densities (2F_obs_−F_calc_) are shown as gray mesh at σ = 2.5 (WT) and σ = 1.7 (A16/20G mutant). Membrane borders are indicated as gray bars, cytoplasmic and periplasmic sites are labeled with C and P, respectively.

Mutations that reduce the number of alanine residues in the 16AxAxAVA22 motif of alkaliphilic *B. pseudofirmus* OF4 by replacing them with glycine residues were found to increase the mobility of the *c-*ring on SDS-PAGE when the first alanine (A16) was among the residues that was replaced (Liu et al., 2011). The A16 residue was shown to have more hydrophobic interactions than the other three alanines in a network in which the alanine methyl side chains interact with atoms from other residues (Preiss et al., 2013). Mutants were constructed in which either a single A16G mutation or a double A16G/A20G mutation were present. The single mutant displayed two bands on SDS-PAGE, suggesting that both a significant amount of *c-*ring with WT stoichiometry and *c-*ring with a lower than WT stoichiometry was present (Liu et al., 2011). The *c*-ring of the double mutant, showing only the faster migrating band, putatively had a lower stoichiometry. Subsequent atomic force microscopy (AFM) studies showed the presence of a mix of 12- and 13-subunits in the single mutant and of 12-subunits in the double mutant (Preiss et al., 2013). As confirmed by X-ray crystallography, the WT *c-*ring has 13-subunits, while the A16G/A20G double mutant displays a 12-subunit ring (Figure 6). Changing the stoichiometry from 13 to 12 resulted in a *c*-ring with a significantly smaller circumference and diameter (Figure 6). In support of these reported structural changes, the molar growth yield of the single A16G mutant was modestly reduced at pH 10.5 while that of the double mutant was reduced by almost 50% (Preiss et al., 2013).

**Figure 7:**
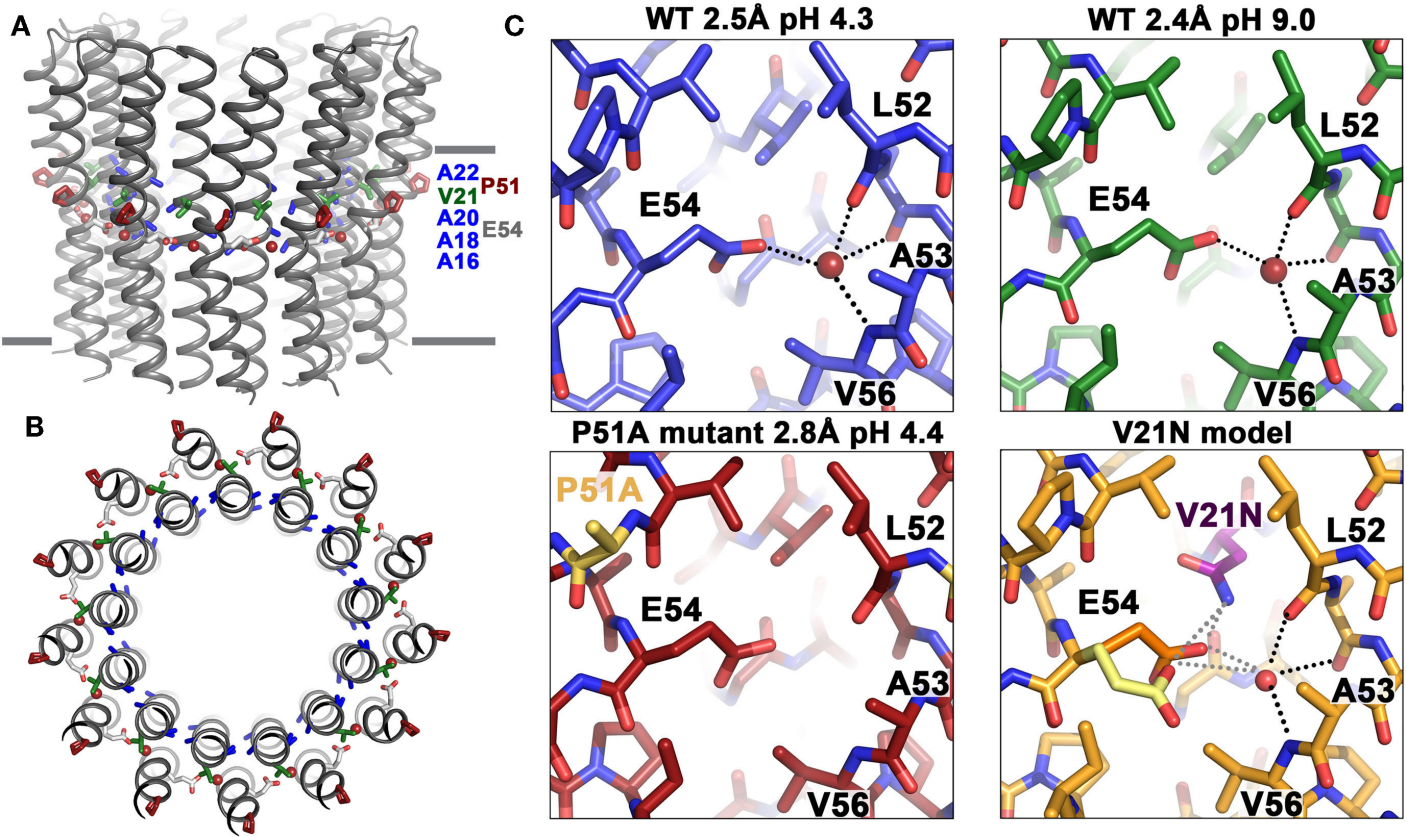
**Structural adaptations of the *B. pseudofirmus* OF4 *c*-ring to an alkaliphilic lifestyle**. The *c*-ring of *B. pseudofirmus* OF4 is shown **(A)** in side view, perpendicular to the membrane and **(B)** in top view. Amino acids that were found to be adapted to the alkaliphilic lifestyle are highlighted in blue (alanine motif), red (Pro51, from the PxxExxP motif), and green (Val21, within the alanine motif AxAxAVA). Membrane borders are indicated by gray bars. **(C)** X-ray crystallographic structures of the *B. pseudofirmus* OF4 *c*-ring ion-binding sites at pH 4.3 (blue), at pH 9.2 (green), the mutant P51A (red), and the mutant model V21N (orange) (Preiss et al., 2010, 2014). H-bonds around the conserved E54 residue (responsible for reversible H^+^ binding, see text) are indicated by dashed lines. The water molecule in the ion-binding site is shown as a red sphere. The V21N model shows E54 in *I. tartaricus*-like conformation (1yce, yellow, Meier et al., 2005) in addition to the WT *B. pseudofirmus* OF4 conformation (orange). The *I. tartaricus*-like conformation might be energetically favorable in the V21N mutant due to the formation of an additional H-bond between N21 and E54 [for details see Preiss et al. (2014)].

Studies of the ion-binding site of the *c*-subunit of *B. pseudofirmus* OF4 have revealed features that are clearly adaptations to alkaliphily. A side view and a top view of the rotor ring is shown in Figures 7A,B, respectively. In Figure 7C (top two panels), binding sites of wild-type (WT) *c-*subunit structures that were crystallized at different pH values; and below them, the consequences of a P51A mutation of the 51PxxExxP motif (lower left) and a V21-to-N mutation in the 16AxAxAVA22 motif (lower right) are shown (Preiss et al., 2014). The top two panels of Figure 7C show that the structure of the ion-binding site is essentially identical in crystals that were prepared at pH 4.4 vs. pH 9.0, highlighting the high affinity H^+^ binding propensity of the c-ring over a wide pH range. The structure of the P51A mutant binding site illustrates the role of the first proline of the PxxExxP motif of the C-terminal *c-*subunit helix in binding a water molecule in the ion-binding site of each subunit. The retention of the water molecule depends upon the presence of P51 and appears to play an important role in preventing proton loss from the ion-binding site during rotation. The V21N mutated *c*-ring had to be modeled since its instability prevented the determination of its crystal structure. The final panel illustrates subtle but important changes that significantly compromise the ion-binding E54 WT conformation upon mutation of valine (conserved in alkaliphiles) to asparagine (conserved in neutralophilic *Bacillus* species). The coordination network of the ion-binding site slightly changes as a result of this mutation, impacting the proton-binding affinity of E54, in agreement with the observed impaired growth phenotype on malate medium at pH 10.5 (Preiss et al., 2014).

In contrast to the scenario with aerobic alkaliphiles that couple *c-*rotor rotation exclusively to proton movements, it is worth noting that some anaerobic alkaliphiles, which are fermentative and do not carry out oxidative phosphorylation, can couple their ATPase (in this case a hydrolase) to pump sodium ions uphill against the trans-membrane ion gradient. This property was exemplified by the rotor ring of *Clostridium paradoxum*, which was found to be coupled to sodium ions and which has an undecameric (*c*_11_) rotor ring (Meier et al., 2006).

### Strategies that may help resolve the thermodynamic challenge of proton-coupled ATP synthesis at high pH

**Figure 8:**
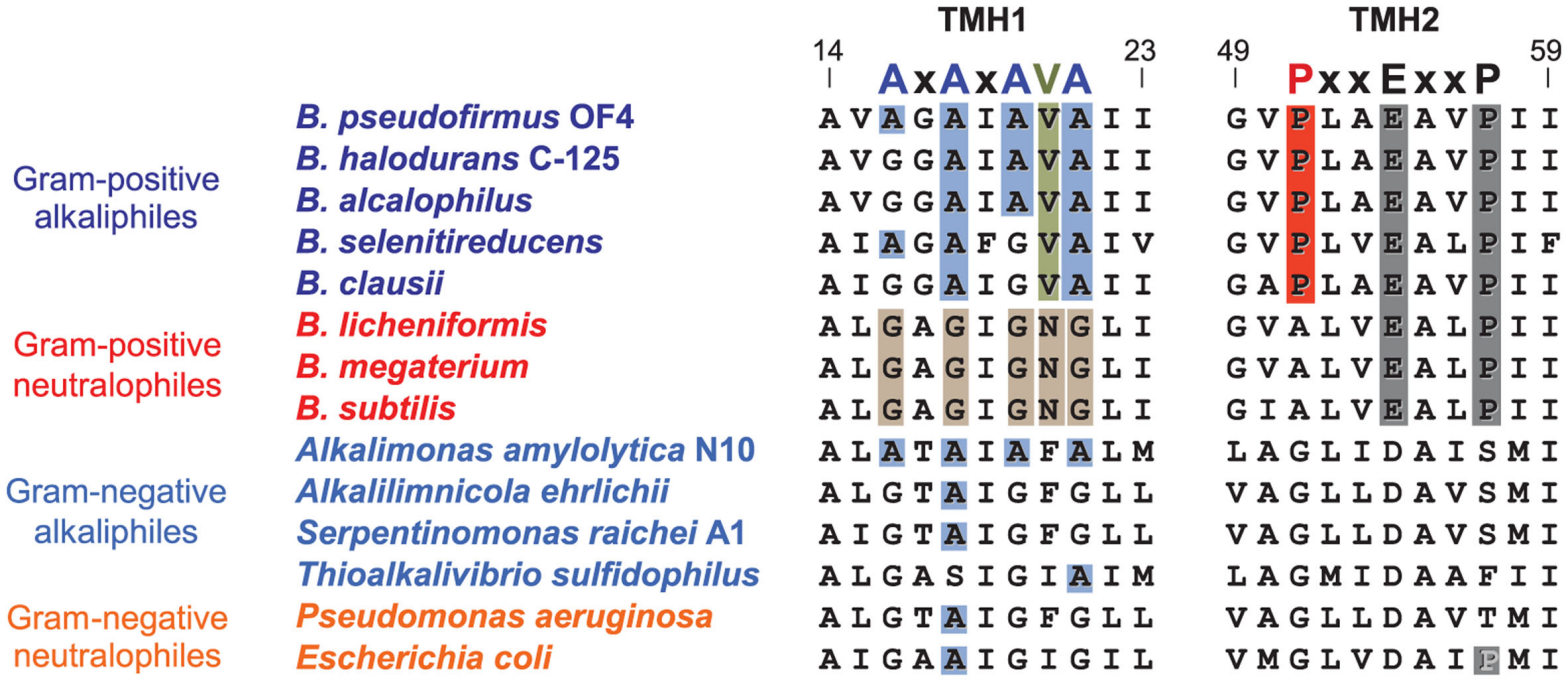
**Alignment of the key regions of TMH1 and TMH2 of different *c*-rings contrasting the features of the Gram-positive *Bacillus* alkaliphiles with the sequences of Gram-negative alkaliphiles**. The names of Gram-positive alkaliphiles are shown in blue, while the Gram-negative alkaliphiles are in light blue. In addition, Gram-positive neutralophiles are in red and Gram-negative neutralophiles are in orange. The TMH1 alkaliphile motif AxAxAx(V)A at positions 16 through 22 (*B. pseudofirmus* OF4 numbering) is shown in blue, and V21 between the last two alanines is shown in green. These features are mostly absent in Gram-negative alkaliphiles, with the notable exception of *Alkalimonas amylolytica* N10, whose sequence is AxAxAxA, i.e., similar to *B. pseudofirmus* OF4, except for the absence of V21. None of the Gram-negative alkaliphiles has the motif of *Bacillus* alkaliphiles in TMH2, PxxExxP, where the proline (P51 in *B. pseudofirmus* OF4) unique to alkaliphiles is shown in red. Yet these Gram-negative alkaliphiles are expected to be proton-coupled, lacking the Na^+^-binding residues of *I. tartarticus* (see Figure 5).

After proton-coupling of alkaliphile ATP synthases was clearly established, a major focus was set next on elements and hypotheses that rationalize the thermodynamic challenge of an apparently insufficient bulk PMF to account for the observed synthesis. Much of the effort and hypotheses have focused on rapid movement of protons from the proton-pumping complexes to the proton-coupled ATP synthase. Studies in support of a more localized movement near the membrane surface included observations by Heberle of proton transfer across bacteriorhodopsin that then extended along the membrane (Heberle, 2000). Proposals of near-membrane barriers to proton equilibration with the bulk phase include compartmentalization and aspects of proton-electrostatics (Mulkidjanian et al., 2006; Liu et al., 2007; Ojemyr et al., 2010; Sandén et al., 2010; Lee, 2012; Wilkens et al., 2013; Rieger et al., 2014). These proposals all have a major focus on the near-membrane environment. However, a comparison of the sequences of *c*-subunits from Gram-negative alkaliphiles with those from Gram-positive alkaliphiles, as shown in Figure 8, suggests that there may be additional cellular factors besides F_o_ adaptations that may have roles in maintaining pumped protons in the periplasm or, perhaps, in some instance, enhancing the ability of a particular strain to maintain a lower periplasmic pH. The alignment in Figure 8 includes examples of both Gram-positive and Gram-negative aerobic alkaliphiles as well as neutralophiles from both groups. It is striking that none of the Gram-negative alkaliphiles requires any of the C-terminal PxxExxP motif adaptations within the *c*-ring that are crucial in Gram-positive alkaliphiles. Moreover, only *Alkalimonas amylolytica* N10, an aerobic Gram-negative, NaCl-dependent alkaliphile isolated from Lake Chahanor in China (Ma et al., 2004) has the suite of four N-terminal alanines, but without the V21 that plays an important role in alkaliphilic *Bacillus* strains. It will be of interest to examine whether the *c-*ring of *A. amylolytica* N10 has a higher *c*-subunit stoichiometry than those of the other Gram-negative alkaliphiles shown, all of which have only a single N-terminal helix alanine. It is also notable that *Serpentinomonas raichei* A1 grows in an unusually high pH environment, even for alkaliphiles (pH 11.6), without features that are critical to alkaliphilic *Bacillus* strains (Suzuki et al., 2014).

**Figure 9:**
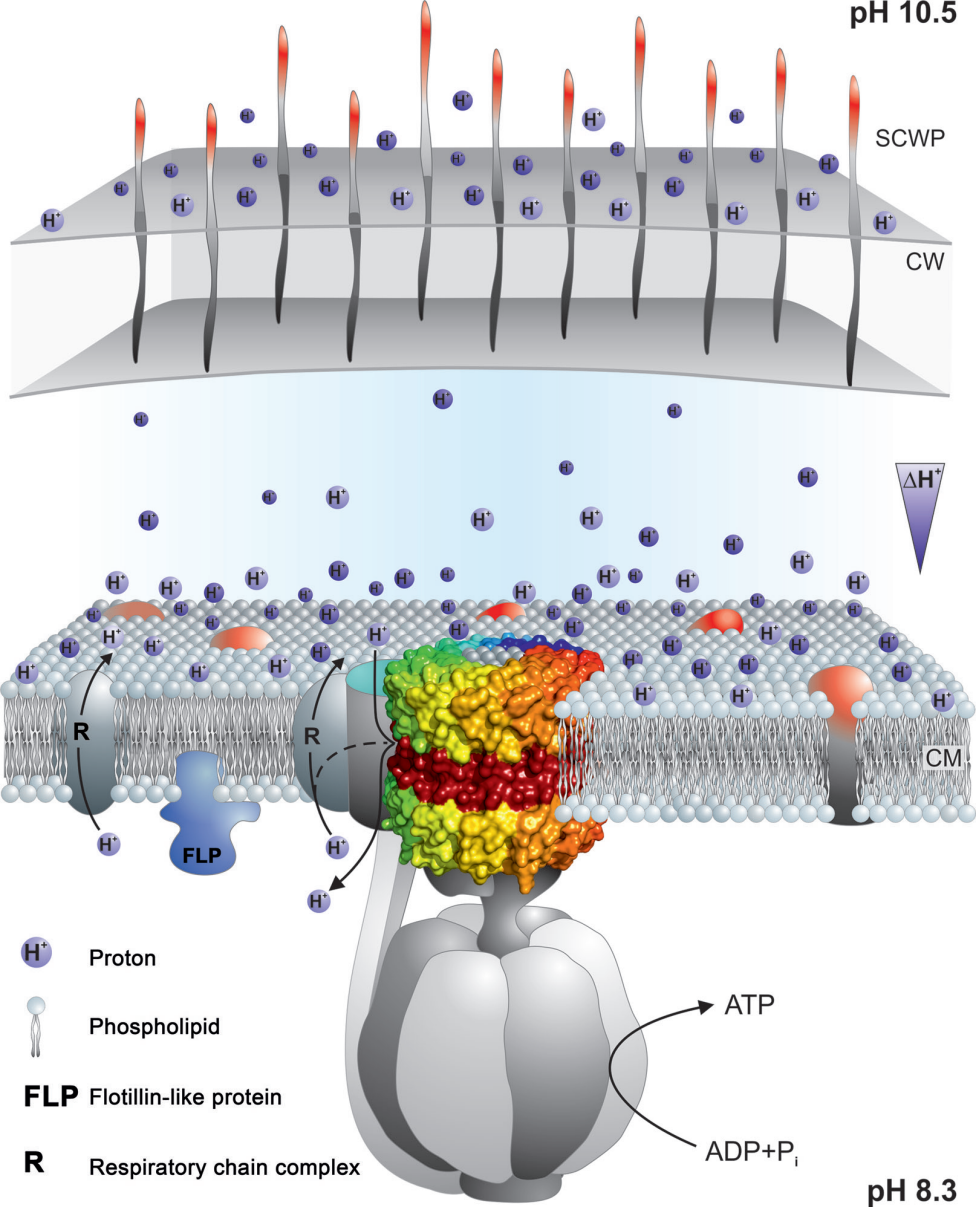
**Summary of properties that may contribute to a delayed loss of protons pumped by the respiratory chain to the bulk medium outside the cell**. The F_1_F_o_-ATP synthase is shown embedded in the cytoplasmic membrane with a proton-pumping respiratory chain element near the synthase. Nearness might be supported by properties of the phospholipids that facilitate rapid movement of protons on or near the membrane surface and/or by an innate tendency of some respiratory complexes to form super-complexes to enhance electron transport and its attendant proton-pumping. Micro-domains in the coupling membrane, such as those attributed to the presence of flotillins, may contribute to compartmentalization. In addition, the cell wall (CW) and secondary cell wall layers (SCWP) may provide a significant barrier, especially in Gram-negative bacteria that have outer membranes as well as other SCWPs, but also in Gram-positive bacteria such as alkaliphiles with multiple, charged SCWPs that could form a significant barrier. For more detailed explanations, see the text. The observation of reduced capacity for alkaliphily in *Bacillus halodurans* C-125 and *Bacillus pseudofirmus* OF4 when negatively charged SCWPs are lost by mutation, is suggestive of some proton trapping on the outside surface of cell wall. This is indicated by the protons shown to be associated with that surface.

The Gram-negative alkaliphiles are likely to be protected by the outer membrane that they possess, and perhaps the outer membrane of mitochondria should be considered for playing at least some role in delaying pumped proton equilibration with the bulk phase or cytoplasm. As shown in Figure 9, there are additional strategies that may contribute to delays in proton equilibration with the bulk phase. It has been suggested that particular phospholipids might play a role, and specifically the high cardiolipin level of *B. pseudofirmus* OF4 might delay proton equilibration (Haines and Dencher, 2002), but at least in *B. pseudofirmus* OF4, mutants lacking detectable cardiolipin were not impaired in non-fermentative growth at high pH (Liu et al., 2014).

Other delays in proton equilibration might involve the entire periplasmic space, which was shown in Gram-positive bacteria by Matias and Beveridge (2005) is surrounded in bacteria by cell wall layers and additional layers of various secondary cell wall polymers that are often highly charged (Takami, 2010). In *B. pseudofirmus* OF4, mutational loss of the S-layer gene *slp* results in a significant deficit in non-fermentative growth at the upper pH range and the S-layer itself is only one of several SLH-motif polymers that contribute to charges on the outside surfaces. Additional contributions are made by charged carbohydrate polymers and a poly-glutamic acid polymer (Gilmour et al., 2000; Janto et al., 2011). In alkaliphilic *B. halodurans* C-125, the polymers are different but also appear to be candidates for delaying proton equilibration. The polymers in the *B. halodurans* C-125 surface include teichuronopeptides, teichuronic acids, and poly-glutamate and poly-glucuronate that are proposed to impact very significantly on the actual pH that this alkaliphile can maintain in the periplasm (Aono, 1987; Tsujii, 2002).

Further elements, that are constituents of the membranes themselves, should also be considered in connection with retention of protons at near the membrane surface. Carotenoids have been found to lend the yellow color to many alkaliphile membranes and it is possible that carotenoids might impart properties to the cytoplasmic membrane that enhance closeness of proton pumps and synthases, in addition to their likely role in scavenging reactive oxygen species (Aono and Horikoshi, 1991; Steiger et al., 2015).

Finally, even stronger candidates for promoting micro-domains that might bring the proton pumps into greater proximity with the ATP synthase are membrane flotillins and their associated proteins (Lopez and Kolter, 2010; Bach and Bramkamp, 2013, 2015; Bramkamp and Lopez, 2015). *B. halodurans* C-125 has an alkali-inducible flotillin-T (Zhang et al., 2005) and *B. pseudofirmus* OF4 has a flotillin-A candidate as well as several NfeD protein candidates that are associated with flotillins. It will be of interest to see whether mutational loss of any of these diminishes the alkaliphilic capacity of these alkaliphiles.

## Conclusion

Alkaliphilic bacteria contribute substantially to numerous technologies, some traditional and others at the cutting edge of new applications of alkaliphile products or their cellular capacities for decontamination of specific commercial settings. Alkaliphiles also are of ecological interest as they are part of serpentinizing sites. The finding that alkaliphilic aerobes that grow non-fermentatively, use energy derived from a proton-pumping respiratory chains and complete oxidative phosphorylation using proton-coupled ATP synthases has challenged a tenet of the formal Mitchellian chemiosmotic hypothesis. Rather than focusing only on narrow solutions to the thermodynamic problem that this re-consideration raises, it is worth considering the broader physiology of alkaliphiles, which may have evolved numerous adaptations that together contribute to their extremophilic capacities.

## Author Contributions

All five authors participated in planning aspects of the manuscript, provided critical advice about the organization and content during the drafting, which was done by TK, and reviewed the submitted version. LP produced Figures 6, 7, and 9, with input from TM and TK, SS contributed the images for Figure 1 and critical advice and expertise on the serpentinization sections, and DH produced Table 1 and Figures 5 and 8, and drafted Figures 2 and 3 as well as the figure legends with TK.

## Conflict of Interest Statement

The authors declare that the research was conducted in the absence of any commercial or financial relationships that could be construed as a potential conflict of interest.
